# Experiences and perception towards reproductive health education among secondary school teachers in South India

**DOI:** 10.1186/s12978-021-01224-6

**Published:** 2021-08-26

**Authors:** Nitin Joseph, Vaibhav Mahato, Akhil Pandey, Shikha Mishra, Garima Prakash, Rishika Gandhi

**Affiliations:** 1 Department of Community Medicine, Kasturba Medical College, Mangalore, Manipal Academy of Higher Education, Manipal, Karnataka India; 2 Kasturba Medical College, Mangalore, Manipal Academy of Higher Education, Manipal, Karnataka India

**Keywords:** Experiences, Perception, Reproductive health education, Secondary school teachers, Urban area

## Abstract

**Background:**

Reproductive health education (RHE) is an important component of school curricula. It helps students in the decision-making process regarding several issues concerning reproductive health. However delivering RHE at schools is a difficult task for the teachers.

**Methods:**

This study was conducted to assess the experiences and perceptions towards reproductive health education (RHE) among 236 secondary school teachers in January 2019. Data were collected using a self-administered questionnaire.

**Results:**

Only 21 (8.9%) were trained in RHE. Majority [179 (75.8%)] identified cultural barriers as the major challenge involved in its implementation. 95 (40.3%) teachers felt that the provision of sexual education as a part of RHE will promote pre-marital sexual activity among the students. Of the total, 185 (78.4%) had average while 51 (21.6%) participants had a good perception towards RHE. It was taught in only 3 (16.7%) out of the 18 schools surveyed. Only 11 (4.7%) participants felt that the availability of teaching aids to conduct RHE classes at their schools was adequate. Hardly 14 (5.9%) teachers had taken RHE classes for students. Among the rest, 135 (60.8%) expressed their willingness to take RHE classes with appropriate training. In multi variable analysis, participants aged ≤ 40 years (*p* = 0.031), those belonging to nuclear families (*p* = 0.013), and those who had taken classes in RHE (*p* = 0.037) had significantly good perception level towards RHE.

**Conclusions:**

Teachers therefore need to be trained and given more opportunities to take RHE sessions which will help improve their perception towards RHE. Schools need to be better equipped with resources and various perceived barriers need to be overcome before RHE can be successfully implemented.

**Supplementary Information:**

The online version contains supplementary material available at 10.1186/s12978-021-01224-6.

## Plain language summary

This study was conducted to assess the experiences and perceptions towards reproductive health education (RHE) secondary school teachers. The participants provided the required information by filling a questionnaire. Hardly one in ten of them had prior training in RHE and one in twenty had taken RHE classes at schools. More than three-fourth of them felt that cultural barriers could pose problems in its implementation at schools. One in four teachers had good perception towards RHE. Two in three among teachers, who had not taken RHE classes before, expressed their willingness to take RHE classes with appropriate training. Favourable perception towards RHE were expressed by teachers who were young, from small families and those who had taken RHE classes before.

**Supplementary Information:**

The online version contains supplementary material available at 10.1186/s12978-021-01224-6.

## Introduction

Reproductive health education (RHE) is an important component of school curricula. It helps students in the decision-making process regarding several issues concerning reproductive health [1, 2]. The international community has always lent its support for the implementation of RHE at schools thereby protecting the rights of the adolescent population [3]. The importance of RHE has been acknowledged in the Sustainable Development Goals Agenda so as to ensure that the necessary knowledge and skills in this area are acquired by all learners. This would support the efforts aimed at ending all forms of violence against girls and women everywhere [4].

The scenario of its implementation in schools in developing countries like India has not been fully explored within academic literature. The Adolescence Education Program (AEP) in India was launched in 2005 to cover all secondary schools [5]. However, several political, religious leaders and teachers themselves opined that the AEP was against Indian cultural and moral values. Critics also felt that its introduction might encourage sexual activity among adolescent population. It was therefore banned across several states in India, including the state of Karnataka in 2007 [6–8].

The current state of RHE in schools across India appears to be in a disorganized manner. Most teachers do not teach RHE due to reasons such as embarrassment, or it not being part of the curriculum [9]. Studies have also observed that most parents are hesitant to discuss reproductive health-related issues with their children [10]. Moreover the information on these matters obtained from mass media and society, although easily accessible, are not always accurate and reliable.

Family, society and schools all have a responsibility in providing RHE to the adolescent population. Since teachers spend a considerable amount of time with the students, it is easier for them to implement RHE as a part of teaching activity. Hence, schools become an ideal and reliable setting to offer RHE for the young population [11]. For this to materialize, the teachers need to be first equipped with the necessary knowledge, skills and comfort level to effectively deliver RHE. For effective implementation of sessions on any sensitive topics such as RHE, the concerns and the expectations desired by teachers for teaching RHE in the classroom set-up, needs to be well understood.

This study was hence designed to study the experiences and perceptions towards RHE among secondary school teachers.

## Methods

This cross-sectional study was conducted in Mangalore city situated on the western coast of South India in January 2019. The Institutional Ethics Committee granted ethical clearance. Adopting a simple random sampling technique, six secondary schools (8th to 10th standard) each from government, aided (institute owned by private management but receives aid from the government) and private schools situated within the city limits were chosen for this study.

The permission to conduct the study at the government and the aided schools was taken from the Block Education Officer (BEO) of Dakshina Kannada District. Further, permission to conduct the study at the school was taken from the respective school principals. Later the school teachers were approached and were informed of the nature and the purpose of the study. The school principals and teachers were assured complete anonymity of the information which were to be collected. Informed consent for participation was taken in writing from all the consenting teachers.

Based on the findings of a previous study done in Chandigarh, India [12] where 88% of school teachers were reported to have favorable perception towards RHE; the sample size using the formula Z_α_^2^pq/d^2^ at 95% confidence intervals, 5% relative precision and adding a non-response rate of 10%, was calculated as 231.

Teachers were approached in their waiting rooms at schools. They were enrolled using the convenience sampling method. Teachers with a minimum of one-year teaching experience and those consenting for participation were included in this study. Data were collected using a self-administered questionnaire. The questionnaire was content validated with the help of subject experts. In the government and aided schools, the Kannada version of the questionnaire was used. It was language validated by translation and back translation with the help of language experts.

Pre-testing of the questionnaire was done based on the responses of 10 teachers chosen non-randomly from a private school which was not included in the main study. Cronbach’s alpha value for the reliability of the questionnaire (after excluding the sociodemographic information of the teachers) was 0.88.

The questionnaire was semi-structured with both open and closed-ended questions. The initial part of the questionnaire was designed to obtain sociodemographic information and details of prior training in RHE amongst the teachers. Section A consisted of questions assessing the views and opinions of teachers regarding RHE. This included various aspects like their perceptions on the necessity of RHE at schools, regarding topics to be covered under RHE, right class to introduce RHE, whether these classes need to be taken separately for boys and girls, need of permanent personnel at schools to teach RHE, barriers involved in teaching RHE at schools and likely problems if there were no RHE for school students. Additionally, teachers’ perception towards a sensitive question, namely, whether the provision of sexual education as a part of RHE at the school level would promote early sexual activity among the learners, was also enquired under this section.

Section B focused on implementation of RHE at the surveyed schools. Teachers in this section were enquired about, the details of personnel taking RHE classes, the availability of teaching aids to conduct RHE and the adequacy of the content related to RHE taught at their schools.

Section C consisted of questions to teachers to enquire their experiences of teaching RHE, the comfort level experienced by them whilst teaching, incidents of any disruptive behavior by students during sessions and any report of objections by the parents for teaching RHE at schools. The various topics under RHE for which the teachers felt that they needed more training were also enquired. For those teachers who had not taken any RHE classes so far, their willingness to teach RHE, if the required training was offered to them, was enquired from them.

The questionnaires took approximately 20 min for each respondent to fill. The investigators were present at the venue to respond to any clarifications from the study participants during the data collection phase. Perception level towards RHE was assessed based on the responses to seven questions designed in a five-point Likert scale which comprised of five positively worded questions: whether teachers felt RHE is necessary for school students, should RHE be placed as a separate chapter in science textbooks, should RHE classes be taken separately for boys and girls, whether there is a need for permanent personnel to be employed at schools to exclusively deal with problems on reproductive health among students and whether they were willing to take classes on RHE with appropriate training. Five points were awarded for a “strongly agree”, 4 for “agree”, 3 for “neutral”, 2 for “disagree” and 1 for “strongly disagree” response. In lieu of the last question, for those teachers who had already taken RHE classes earlier, 5 points meant for the “strongly agree” response, were awarded to each of them. For the other two questions which were negatively worded: whether RHE classes should be taken by same-gender teachers to the same-gender students and whether sexual education will promote early sexual activity among students, reverse scoring was done. The minimum attainable score based on the responses to these seven questions was 7 and the maximum was 35.

Therefore, scores ranging from 7 to 16 were considered as poor, 17 to 26 as average and 27 to 35 as good perception level towards RHE among the teachers.

Data entry and analysis were done using International Business Machines Corporation (IBM) Statistical Package for the Social Sciences (SPSS) for Windows version 25.0, Armonk, New York. Fisher's exact test was used to test association. Chi-square test and Binary logistic regression analysis were also used to determine the variables associated with good perception towards RHE among school teachers. The *p* < 0.05 was taken as the cut-off for statistical significance.

## Results

A total of 257 teachers were eligible to take part in this study. However, only 236 (91.8%) of them returned satisfactorily filled questionnaires.

The mean age of the teachers was 40.3 ± 9.5 years. (Table 1) Only 60 (25.4%) taught science-related subjects while the rest taught other subjects. The mean years of teaching experience among the participants were 11.9 ± 7.1 years. The years of teaching experience ranged from 1 to 33 years. (Table 2).

**Table 1:** Socio demographic distribution of school teachers (n = 236)

Characteristics	Number	Percentage
Age (years)		
≤ 25	7	3.0
26–30	39	16.5
31–35	37	15.7
36–40	40	16.9
41–45	46	19.5
46–50	29	12.3
51–55	14	5.9
> 55	24	10.2
Gender		
Males	88	37.3
Females	148	62.7
Type of family		
Nuclear	138	58.5
Joint	98	41.5
Native place		
Urban	189	80.1
Rural	47	19.9
Total	236	100.0

**Table 2:** Distribution of teachers based upon school related characteristics (n = 236)

Characteristics	Number	Percentage
Type of school (based on ownership)		
Government	80	33.9
Aided	81	34.3
Private	75	31.8
Type of school (based on co-education status)		
Co-educational school	227	96.2
All-boys school	3	1.3
All-girls school	6	2.5
Educational background		
Science	93	39.4
Arts	143	60.6
Subjects taught at school		
Science related	60	25.4
Others	176	74.6
Teaching experience (years)		
1–5	53	22.5
6–10	71	30.1
11–15	46	19.5
16–20	35	14.8
21–25	21	8.9
> 25	10	4.2
Total	236	100.0

Only 21 (8.9%) teachers were trained in RHE. Overall, the training in RHE was observed to be inadequate among the participants. (Table 3).

**Table 3:** Characteristics related to reproductive health education (RHE) training among school teachers

Characteristics	Number	Percentage
Trained in RHE (n = 236)		
Yes	21	8.9
No	215	91.1
Number of training sessions attended (n = 21)		
1	8	38.1
2	10	47.6
3	3	14.3
The time gap between the most recent training session with the present time (n = 21)		
≤ 2 years	4	19.1
2.1–3 years	5	23.8
3.1–5 years	3	14.2
5.1–10 years	4	19.1
> 10 years	5	23.8
Personnel who conducted the most recent training (n = 21)		
Medical professionals	15	71.4
Teachers	6	28.6
The venue of training (n = 21)		
At the school	21	100.0
Certified training (n = 21)		
Yes	2	9.5
No	19	90.5
Other sources of information about RHE (n = 236)^†^		
Textbooks	183	77.5
Internet	141	59.7
Television	137	58.0
Colleagues	119	50.4

^†^Multiple responses

As many as 215 (91.1%) of the total participants agreed/strongly agreed that there was a necessity for RHE for school students. Overall, the teachers had a favorable perception of RHE. (Table 4).

**Table 4:** Perception regarding reproductive health education among school teachers

Characteristics	Number	Percentage
The necessity of RHE for school students		
Strongly agree	67	28.4
Agree	148	62.7
Neutral	14	5.9
Disagree/strongly disagree	7	3.0
When should it be introduced for school children (n = 215)		
1st to 5th standard	23	10.7
6th to 7th standard	88	40.9
8th to 10th standard	104	48.4
When should it be introduced if not during schooling years (n = 7)		
During pre-university course	6	85.7
Before marriage	1	14.3
RHE should be introduced to which gender		
Both boys and girls	222	94.1
Only girls	14	5.9
Should RHE sessions be taken separately for boys and girls		
Strongly agree	82	34.8
Agree	92	39.0
Neutral	14	5.9
Disagree	42	17.8
Strongly disagree	6	2.5
The same gender teacher should teach RHE to the same gender students		
Strongly agree	13	5.5
Agree	65	27.5
Neutral	70	29.7
Disagree	73	30.9
Strongly disagree	15	6.4
Suitable teaching aids to conduct RHE at schools^†^		
Posters	130	55.1
Flip charts	112	47.5
Video films	91	38.6
Models	66	28.0
RHE should be a separate chapter in science textbooks		
Strongly agree	48	20.3
Agree	108	45.8
Neutral	48	20.3
Disagree	31	13.2
Strongly disagree	1	0.4
RHE classes to be taught after usual teaching hours at schools		
Yes	29	12.3
No	142	60.2
Not sure	65	27.5
Should curriculum makers take teacher’s suggestions while preparing a RHE module		
Yes	230	97.5
No	6	2.5
Reasons for the same (n = 230)		
Teachers directly deal with students	69	30.0
Teachers understand students the best	7	3.0
Need for permanent personnel at schools to exclusively deal with reproductive health-related problems among students		
Strongly agree	15	6.4
Agree	106	44.9
Neutral	98	41.5
Disagree	12	5.1
Strongly disagree	5	2.1
Provision of sexual education as a part of RHE will promote premarital sexual activity among the students		
Strongly agree	7	3.0
Agree	88	37.3
Neutral	72	30.5
Disagree	67	28.4
Strongly disagree	2	0.8
Total	236	100.0

^†^Multiple responses

When the teachers were asked regarding who they felt were the right persons to teach RHE to the students, the majority [156 (66.1%)] stated biology teachers. The other personnel identified by participants suitable for this task were student counsellors [88 (37.3%)], obstetricians [83 (35.2%)], any trained personnel [83 (35.2%)], medical officers [66 (28%)], teachers [47 (19.9%)], pediatricians [41 (17.4%)], parents [38 (16.1%)], class teachers [29 (12.3%)], senior teachers [10 (4.2%)] and school principals [10 (4.2%)]. Reasons for these preferences were: due to their proficiency in knowledge regarding reproductive health [223 (94.5%)], their accessibility [32 (13.6%)] and familiarity [25 (10.6%)] with the students, as stated by the participants.

The common topics under RHE to be covered at schools as opined by the participants were concepts of puberty [209 (88.6%)], awareness of good/bad touch [177 (75%)], menstrual hygiene [174 (73.7%)], information on sexually transmitted diseases (STDs) [159 (67.4%)], description and functions of reproductive organs [143 (60.6%)], benefits of ideal family size [140 (59.3%)], information about right age at marriage [139 (58.9%)], concept of menarche [136 (57.6%)], information about right age at pregnancy [134 (56.8%)], sexual abuse/harassment [133 (56.4%)] and about contraceptives [38 (16.1%)].

Topics under RHE which the participants specifically suggested to be introduced before secondary school were: awareness of good/bad touch [55 (23.3%)], about concepts of puberty [20 (8.5%)], description and functions of reproductive organs [13 (5.5%)] and menstrual hygiene [6 (2.5%)].

Topics under RHE which the participants specifically suggested to be introduced after secondary school were: issues concerning teenage pregnancies [48 (20.3%)], about contraceptives [33 (14%)], information on STDs [5 (2.1%)] and description and functions of reproductive organs [5 (2.1%)].

The common challenges involved in teaching RHE in schools as opined by teachers were: cultural barriers [179 (75.8%)], parental objections [94 (39.8%)], lack of a standardized teaching module [61 (25.8%)], teachers not being trained in RHE [52 (22%)], school administrators not recognizing the importance of RHE [15 (6.4%)] and unavailability of sufficient resource materials at schools to conduct RHE sessions [ (11 (4.7%)].

The common problems that would be encountered if there were no RHE at schools as perceived by the participants were: students ending up acquiring incorrect information about reproductive health from various informal sources [109 (46.2%)], students ending up in an anxious state when they encounter issues concerning reproductive health [102 (43.2%)], greater risk of teenage pregnancies [45 (19.1%)], more chances of premarital sexual experiences [39 (16.5%)], more instances of abortions [16 (6.8%)] and risk of having an unsuccessful marital life in future [15 (6.4%)].

Out of the 215 teachers who agreed/strongly agreed with the introduction of RHE in schools, 76 (35.3%) felt that it would help students to understand more about themselves and 29 (13.5%) felt that it would benefit students in getting all their misconceptions cleared regarding this topic. Among the 14 teachers who felt that RHE is to be introduced only for the girls, 6 (42.9%) thought so because, girls in particular need to be aware of consequences following sexual misadventures.

The cumulative perception scores of the participants ranged from 17 to 30. Among them, 185 (78.4%) had average while 51 (21.6%) had a good perception towards RHE.

Implementation of RHE at schools was done in only 3 (16.7%) out of the 18 schools. Implementation of RHE was observed in a government, aided and private school. All these were co-educational schools. Formal RHE classes were given only in the private school and it was for students from 6 to 10th standard. Sessions were taken by both, teachers from the same institute and by teachers from other institutes. In the other two schools, RHE sessions were offered informally for only 10^th^ standard students and the resource persons were teachers from the same institution.

A total of 14 (5.9%) teachers had taken classes on RHE. Overall, the majority of the participants felt that the resource materials for conducting RHE classes at the surveyed schools were not adequate. (Table 5).

**Table 5:** Experiences of teachers with reproductive health education sessions at the surveyed schools

Characteristics	Number	Percentage
Content of RHE delivered at the school		
Adequate	18	7.6
Inadequate	88	37.3
Not sure	130	55.1
Availability of teaching aids at schools to conduct RHE		
Adequate	11	4.7
Inadequate	90	38.1
Not sure	135	57.2
Taken classes on RHE		
Yes	14	5.9
No	222	94.1
If not, willingness to take with appropriate training (n = 222)		
Agree	135	60.8
Neutral	54	24.3
Disagree	12	5.4
Strongly disagree	21	9.5
Topics under RHE for which additional training is required^†^		
Sexually transmitted diseases	56	23.7
Counselling children with issues related to RHE	53	22.5
Physiology of menstruation	21	8.9
Feeling of uneasiness while taking classes on RHE (n = 14)		
Neutral	1	7.1
Disagree	7	50.0
Strongly disagree	6	42.9
Feeling of uneasiness while taking classes on RHE to students of the opposite gender (n = 14)		
Neutral	3	21.4
Disagree	7	50.0
Strongly disagree	4	28.6
Disruptive behaviour by students during RHE sessions (n = 14)		
Yes	2	14.3
No	12	85.7
Parental objection for taking classes on RHE (n = 14)		
Yes	4	28.6
No	10	71.4
Total	236	100.0

^†^Multiple responses

Out of the 21 teachers who underwent training in RHE in the past, 3 (14.3%) had taken classes on RHE for the students. Among the 215 teachers who did not undergo any form of training in RHE, 11 (5.1%) had taken classes on RHE for the students (*p* = 0.116). This also infers that, out of the 14 teachers who had taken classes in RHE, 11 (78.6%) did not undergo any form of training in RHE in the past.

Some of the open suggestions/observations given by teachers regarding reproductive health were: school students often find it uncomfortable in accepting their bodily changes during puberty (5), the current teaching of RHE at schools is inadequate (3), provision of sexual education as a part of RHE may promote premarital sexual activity among the students (3), the mass media have promoted obscenity leading to promiscuous behavior among the students (1) and that there are several misconceptions present regarding menstruation among girls (1).

Participants aged ≤ 40 years, females, those belonging to nuclear families, those who underwent training in RHE and those who had taken classes in RHE, had significantly good perception level towards RHE as compared to the rest (Table 6).

**Table 6:** Association between determinants and perception level towards reproductive health education among school teachers

Characteristics	Perception level towards RHE		Total
	Good no. (%)	Average no. (%)	
Age group			
≤ 40 years	36 (29.3)	87 (70.7)	123
> 40 years	15 (13.3)	98 (86.7)	113
			X^2^ = 8.893, *p* = 0.003
Gender			
Male	12 (13.6)	76 (86.4)	88
Female	39 (26.4)	109 (73.6)	148
			X^2^ = 5.267, *p* = 0.022
Type of family			
Nuclear	40 (29)	98 (71)	138
Joint	11 (11.2)	87 (88.8)	98
			X^2^ = 10.671, *p* = 0.001
Underwent training in RHE			
Yes	9 (42.9)	12 (57.1)	21
No	42 (19.5)	173 (80.5)	215
			X^2^ = 6.143 *p* = 0.013
Taken classes on RHE			
Yes	8 (57.1)	6 (42.9)	14
No	43 (19.4)	179 (80.6)	222
			X^2^ = 11.092, *p* = 0.001
Total	51	185	236

In multivariable analysis, participants aged ≤ 40 years (*p* = 0.031), those belonging to nuclear families (*p* = 0.013) and those who had taken classes in RHE (*p* = 0.037), had significantly good perception level towards RHE as compared to others (Table 7).

**Table 7:** Binary logistic regression analysis of variables associated with good perception level towards reproductive health education among the school teachers (n = 236)

Characteristics	Unadjusted OR	95% CI for unadjusted OR		*p* value	Adjusted OR	95% CI for adjusted OR		*p* value
		Lower	Upper			Lower	Upper	
Age (years)								
≤ 40	2.703	1.386	5.272	0.003	2.147	1.073	4.299	0.031
> 40	1				1			
Gender								
Males	1				1			
Females	2.266	1.114	4.61	0.022	1.014	0.429	2.396	0.975
Type of family								
Nuclear	3.228	1.56	6.679	0.001	2.579	1.219	5.459	0.013
Joint	1				1			
Underwent training in RHE								
Yes	3.089	1.222	7.812	0.013	2.163	0.813	5.751	0.122
No	1				1			
Taken classes in RHE								
Yes	5.55	1.83	16.835	0.001	3.4	1.08	10.709	0.037
No	1				1			

## Discussion

The proportion of teachers who underwent training in RHE was only 8.9% in the present study in comparison to 25.8–70% reported in previous studies [2, 3, 13, 14]. Considering the multidisciplinary nature of RHE, all teachers during their preservice and in-service training years need to be given training in reproductive health.

Close to three-fourth of teachers in the present study felt that RHE sessions need to be taken separately for boys and girls. In co-education RHE sessions, students might experience discomfort as learners of one gender may feel embarrassed to discuss with their teachers, certain topics under reproductive health, in the presence of students of the opposite gender.

More than three-fourth of teachers in this study felt that the cultural barriers were the main challenges involved in teaching RHE at schools. In previous studies, teachers listed religion, culture, restrictive policies, inadequate time being allotted, untrained teachers, lack of confidence among teachers, lack of infrastructure, lack of teaching aids, lack of support from teachers, objections raised by students, parents or school administrators, as the potential barriers for the same [2, 14–20].

A total of 40.3% teachers in this study and 39–71.7% in previous studies felt that sex education as a part of RHE would promote early sexual activity among the students [2, 3, 21]. These misconceptions need to be addressed in training sessions for teachers in order to bring a more favorable perception towards RHE.

Implementation of RHE was seen merely among three of the surveyed schools in the present study. Moreover, just one out of these three schools had sessions taken by teachers from other institutions. In the South African study, it was observed that outside personnel like the Department of Health Officials and school nurses were occasionally invited to take RHE classes for the students [14]. Teachers at these schools felt that this initiative made RHE classes more effective because learners could relate and discuss sensitive issues concerning reproductive health better with outsiders than with their educators [14].

In this study, approximately 8% of respondents felt that the content of RHE sessions and approximately 5% felt that the teaching aids to conduct these sessions were adequate at their schools. Similarly, in the South African study, hardly 10% of teachers felt that the schools had adequate resources to enable them to take RHE classes [14].

In this study, approximately 6% of teachers had taken classes in RHE. Among them, most were not even trained in RHE. This dismal picture was also reported in a study done in four countries of pacific islands where a significant number of teachers taught RHE without any training [2]. Prior research has shown that training has a major influence on the confidence levels of teachers teaching sensitive topics like RHE. Lack of the required training was found to negatively impact their quality of teaching [18, 19].

Among the teachers who had taken RHE classes for students in this study, more than one-fourth of them reported parental objection to the conduct of these classes. In the South African study, 90% of teachers reported a lack of support from parents [14]. Parental counseling on the importance of RHE might help in addressing these issues before initiating RHE sessions at schools.

Certain variables were found to be associated with good perception level towards RHE among participants in this study. In other studies, teachers with more than four years of teaching experience [22], science teachers [22], the gender of the teacher [23], trained teachers [23] and teachers teaching RHE [23] had a more positive attitude towards the importance of sex education at schools.

## Conclusions

It is evident from the findings of this study that few teachers were trained in RHE. Similarly, few teachers had taken classes on RHE. Teachers therefore need to be trained during the pre and in-service training programs and need to be given more opportunities to take RHE sessions. This would benefit them in having a good perception of RHE, as supported by the observations of this study. Schools need to be better equipped with resources for the successful implementation of RHE.

## Limitations

There is a possibility of under reporting of information on this sensitive topic by participants of this study**.**

## Supplementary Information

**Additional file 1.** Raw data of the research study.

## Data Availability

The SPSS spread sheet containing the data of this research study has been uploaded as an Additional file 1.

## Abbreviations

RHE: Reproductive health education
AEP: Adolescence Education Program
BEO: Block Education Officer
IBM: International Business Machines Corporation
SPSS: Statistical Package for the Social Sciences
STDs: Sexually transmitted diseases
CI: Confidence interval
OR: Odds Ratio
